# Drug delivery systems for treatment of temporomandibular joint osteoarthritis

**DOI:** 10.3389/fphar.2022.1054703

**Published:** 2022-11-07

**Authors:** Xinqi Huang, Xuefeng Pan, Xiner Xiong, Zhihe Zhao, Xiao Cen

**Affiliations:** ^1^ State Key Laboratory of Oral Diseases & National Clinical Research Center for Oral Diseases, West China Hospital of Stomatology, Sichuan University, Chengdu, China; ^2^ Hospital of Stomatology, Zunyi Medical University, Zunyi, China

**Keywords:** drug delivery, intra-articular injection, transdermal administration, temporomandibular joint, osteoarthritis

## Abstract

The number of people suffering from temporomandibular joint osteoarthritis (TMJOA) has been increasing. TMJOA cause joint noise, pain on TMJ and/or masticatory muscles, and restricted mandibular movement, which disturb eating, laughing and conversation, and impose serious lifestyle impediments. Chondrocyte apoptosis, extracellular matrix degradation, synovitis, and subchondral bone remodeling are the main pathological features of TMJOA. Various drug delivery systems are developed to controlled release at specific activation sites with high bioactivity and inhibit rapid dilution to enable long-term therapeutic response, which present great potential for the treatment of TMJOA. This review focuses on recently developed drug delivery systems by different administration in the TMJOA treatment, and summarizes their effects, duration, safety, and limitations, which would pave the way for development of TMJOA therapy.

## 1 Introduction

Temporomandibular joint (TMJ) is a synovial joint responsible for complicated mandibular movement and various normal function (e.g., eating, laughing, and conversation). As TMJ is sensitive to mechanical force, excessive loading on normal joint or normal loading on impaired joint could both cause the destruction of joint structure, and eventually progress into TMJ osteoarthritis (OA). Although the etiology of TMJOA is not fully understood, yet several factors are commonly considered, including malocclusion, mental factors, and abnormal maxillofacial muscles. With the three major symptoms of TMJOA (i.e., joint noise, pain, and restricted mandibular movement), the life quality and normal function of patients will be severely affected. Pathologically, cartilage extracellular matrix is degraded by matrix metalloproteinases (MMPs), a disintegrin and metalloprotease with thrombospondin motifs (ADAMTS), and chondrocyte apoptosis in affected cartilage tissue; under the mechanical and inflammatory microenvironment, subchondral bone becomes thinner in the early stage while bone sclerosis and osteophyte formation are in the late stage; synovitis are also the hallmarks of TMJOA and further exacerbate the local inflammatory environment.

Currently, the common drugs introduced to treat OA clinically include glucocorticoids, non-steroidal anti-inflammatory drugs (NSAIDs), viscosupplements, and other traditional drugs through oral administration, intra-articular injections, and/or transdermal patch. However, these methods can only relieve inflammatory symptoms, but barely inhibit the progress of OA or reverse the course and cure OA. Furthermore, the drugs also have the disadvantages of being easily eliminated and the risk of causing adverse reactions.

**FIGURE 1:**
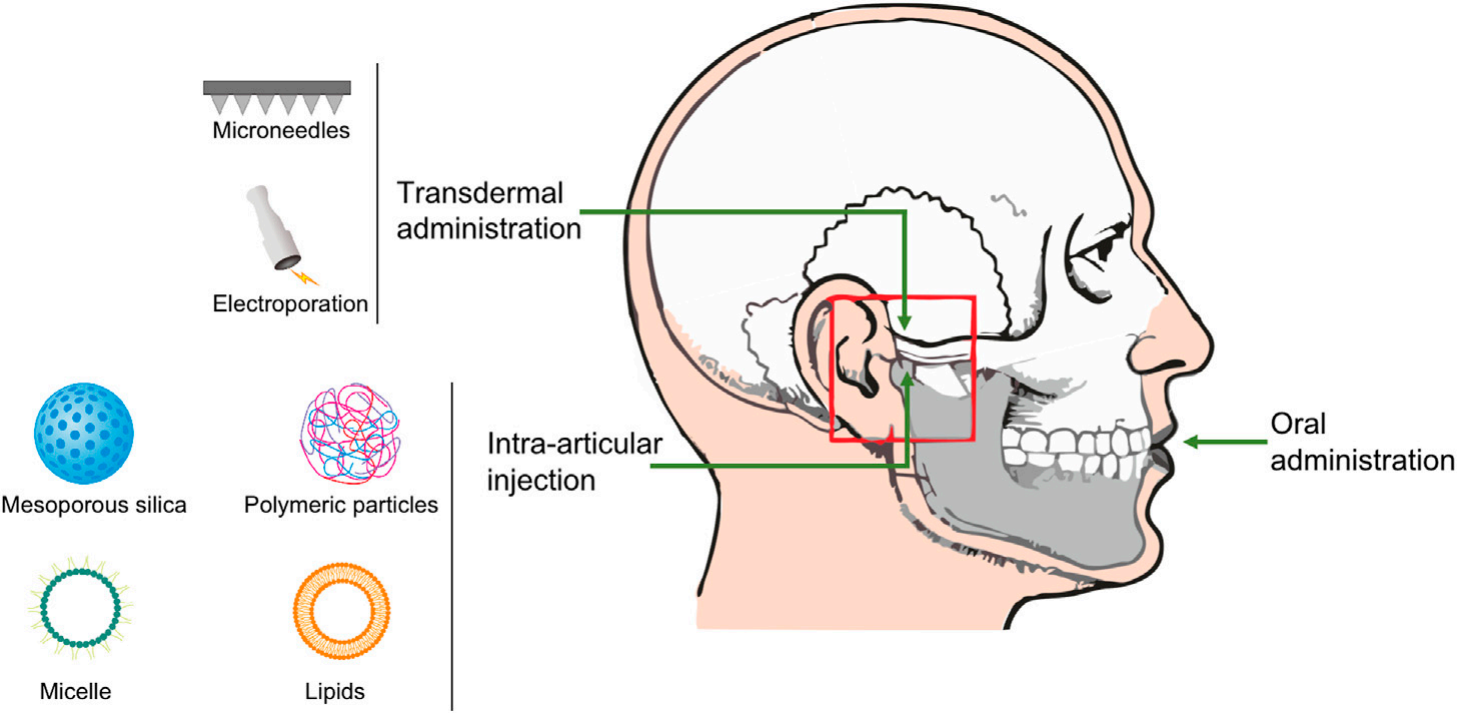
Schematic of various nanoparticles and administration routes in the TMJOA treatment.

Herein, this review focuses on recently developed drug delivery systems by different administration in the TMJOA treatment (Figure 1), and summarizes their effects, duration, safety, and limitations.

## 2 Intra-articular drug delivery systems

IA injection, which delivers drugs to the articular cavity directly, is the most widely used administration mode for the treatment of OA. However, after small- and large-molecule drugs enter the TMJ cavity, they are easily cleaved by synovial capillaries and lymphatics, which results in failure of the drug to reach a therapeutic dose in the articular cavity. Frequent and multiple administrations are utilized to solve this challenge currently, which cause pain, subcutaneous atrophy, increase risk of infection, joint disease progression, and decrease the patient compliance (Ta and Dionne, 2004).

Drug delivery systems can suspend in the joint cavity, slowly releasing the drug into the cavity. In some condition, synovial tissue can retain the drug delivery systems, and the drugs are released directly into the articular capsule and influence immune cells or fibroblasts. Therefore, sustained-release drug delivery systems are needed to extend the residence time to reduce adverse effect caused by frequent injections and large dose, and improve the efficacy of these drugs in the articular cavity. Various drug delivery systems for IA injection have been developed to enhance the bioavailability and prolong the circulation time to improve the efficacy of drugs, which are summarized in this section (Table 1).

**TABLE 1:** Different drug delivery systems for intra-articular injection.

Carrier molecule	Active molecule	Model	Particle size	Effects on TMJOA	Duration	Ref
Lipid, P68	Naproxen	Carrageenan-induced inflammation in rat TMJs	279–591 nm	Downregulate inflammatory cytokines	10 days	Guilherme V. A. et al. (2019)
HA, PLGA	Parecoxib	LPS-induced inflammation on synovial cells, mechanically-induced synovitis in rat TMJs	25.32 ± 1.01 μm	Downregulate inflammatory cytokines	>2 weeks	Zhu D. et al. (2021)
PLGA	15d-PGJ_2_	Formalin-induced TMJ nociception in rats	182–220 nm	Decrease IL-1β release and nociceptive behavioral response	45 min	Clemente-Napimoga J. T. et al. (2012)
PLGA, PEI	FcγRIII siRNA	CFA-induced inflammation in rat TMJs	18 ± 1 μm	Reduce inflammatory cytokines and nociceptive behavioral response	9 days	Mountziaris et al. (2012)
Chitosan	HA	Health TMJs of rabbits	Not reported	Sustained release of HA	7 days	Talaat et al. (2016)
MSN-CC, PEI	HAS2 protein	CFA-induced inflammation and MIA-induced bone defects in rat TMJs	≈175 nm	Maintain the high HAS2 activity, inhibit the synovial inflammation, repair bone defects	3 weeks, 12 weeks	Li et al. (2019)
Poloxamer	15d-PGJ_2_	Formalin-induced TMJ nociception in rats	≈70 nm at 25°C, ≈50 nm at 37°C	Sustained release of 15d-PGJ_2_, reduce inflammatory cytokines and nociceptive behavioral response	14 days	Abdalla et al. (2020)

### 2.1 Lipid nanoparticle systems

Lipid nanoparticles are composed of cationic or neutral lipids, cholesterol, and polyethylene glycol (PEG)-lipids, which simulate cell membranes with a diameter of about 100 nm. Lipid nanoparticles are widely used for non-viral drug delivery, as they have a large number of advantages including high encapsulation efficiency, strong tissue permeability, low cytotoxicity and immunogenicity (Cullis & Hope, 2017).

Owing to the amphoteric properties of lipids, lipid nanoparticle systems can not only encapsulate hydrophilic drugs in the interior aqueous space, such as water-soluble dexamethasone phosphate (Anderson et al., 2010), but also hydrophobic drugs inserted in the hydrophobic region of the bilayer, such as kartogenin (KGN) (Yang et al., 2020). Additionally, diverse lipids species and ratios can be selected to easily fine-tune the physicochemical properties of lipid nanoparticle systems. The outer surface is also capable of functional modifications to attain long-term stabilized and targeted drug-delivery carriers, and achieve diagnostic and therapeutic platforms (Al-Jamal et al., 2011; Namiki et al., 2011).

Naproxen is one kind of nonsteroidal anti-inflammatory drugs (NSAIDs), which serves as the inhibitor of cyclooxygenase-1 (COX-1) and COX-2, and thus naproxen is commonly prescribed for the treatment of TMJOA patients to relieve symptoms and restore function (Cigerim and Kaplan, 2020). Encapsulation of naproxen into lipid nanoparticles designed for administration into TMJ has been successfully reported, which is capable to reduce the administration frequency and improve the therapeutic efficacy (Guilherme et al., 2019). This work develops an optimized nanostructured lipid carrier which is composed of 2% Pluronic^®^ F68 (P68) and 20% total lipids (70/30 solid lipid/liquid lipid), and its encapsulation efficiency of naproxen was 99.8% ± 0.2%. This naproxen-loaded system can limit the leucocyte migration to the inflamed rat TMJs and downregulate inflammatory cytokines levels for 10 days after application (Guilherme et al., 2019). This impressive therapeutic effect suggests the sustained release profile of naproxen provided by the nanostructured lipid carrier can prolong the anti-inflammatory effect after one application, and multiple administrations will not be required, which can increase the patient compliance to the treatment and reduce naproxen side effects. Moreover, the nanostructured lipid carrier is composed of the lipids with good biocompatible nature, and thus it may decrease drug local toxicity and subsequent inflammation of injected sites at TMJ (Tamjidi et al., 2013).

### 2.2 Poly (lactic acid-co-glycolic) acid-based system

Poly (lactic-co-glycolic) acid (PLGA) is a kind of injectable, degradable, and functional polymer organic compound. PLGA has good biocompatibility, performance of encapsulation and film-forming capacity, and thus it is widely applied to the development of new pharmaceuticals and medical engineering materials (Ding and Zhu, 2018). Mountziaris et al. (2010) produced PLGA microparticles by an established double-emulsion solvent extraction technique, and investigated *in vivo* biocompatibility of these PLGA microparticles for intra-articular drug sustained release in the TMJs. Computerized meal pattern showed that bilateral intra-articular injection of 15, 30, or 50 mg/ml PLGA microparticles did not induce TMJ pain or nociception over 6 days. No additional tissue reaction but a mild inflammatory infiltrate was observed in the 15, 30, or 50 mg/ml PLGA groups, compared with injection with carrier solution alone.

Parecoxib is a highly selective inhibitor of COX-2, which suppresses prostaglandin (PG) synthesis and alleviates chronic inflammatory conditions such as OA. Parecoxib-loaded hyaluronic acid (HA)-PLGA microspheres are fabricated for sustained release in TMJ synovitis therapy (Zhu et al., 2021). The loading rate of parecoxib in this delivery system reaches 17.12%–20.95%, while its drug release tests *in vitro* show a slow sustained release over 28 days. Treatment with parecoxib-HA-PLGA microspheres declined inflammatory gene expression in synovial cells *in vitro*, and injection of these microspheres can downregulate expression levels of inflammatory cytokines for more than 2 weeks in rat models. Some studies encapsulated 15d-PGJ_2_, which is a natural ligand for peroxisome proliferators-activated receptor-γ (PPARγ), into PLGA to develop nanocapsules for the therapy of TMJ pain (Clemente-Napimoga et al., 2012). Compared with injection of 15d-PGJ_2_ only, the intra-articular injection of 15d-PGJ_2_-PLGA nanocapsules could decrease the release of IL-1β cytokine, and nociceptive behavioral response was also reduced significantly in the formalin-induced rat models.

After the first therapeutic small interfering RNA (siRNA) (ONPATTRO™) was approved by United States Food and Drug Administration (FDA) in 2018, many biopharmaceutical and biotech companies are developing RNA-based therapies which are in different stages of drug development pipeline (Bai et al., 2020; Kulkarni et al., 2021; J. Yang et al., 2020; Z. Yang et al., 2020). The RNA drugs include short interfering RNA (siRNA), oligonucleotides, messenger RNA (mRNA), microRNA (miRNA), RNA aptamers and so on, and RNA application involves mediating transcriptional activation, encoding disease-related proteins, and regulating expression of specific genes and proteins, which present great potential for the treatment of OA. However, the clinical application of RNA therapeutics remains a challenge. The action sites of RNAs are mostly intracellular, while RNA molecules are hydrophilic and negatively charged as well as have high molecular weights, which make it difficult for RNAs to cross cell membrane into cytoplasm. Additionally, RNAs are sensitive to ubiquitous RNases. It is reported that unprotected RNAs have an extremely short metabolic or systemic half-life (e.g., less than 7 min) (Soutschek et al., 2004). Therefore, delivery systems are critical for the development and application of RNA drugs.

Matrix metalloproteinase 13 (MMP13) can degrade type II collagen which is the vital cartilage structural protein, and si-MMP13 is reported as a disease modifying therapy for OA (Yang et al., 2020). Shape-defined PLGA microplates are established to carry si-MMP13 (Bedingfield et al., 2021; Bedingfield et al., 2021.). It is showed that PLGA-based microplates maintained si-MMP13 in the joint cavity longer significantly, and injection with these microplates against MMP13 maintained potent knockdown of MMP13 gene expression and protein production in the joint tissues over 28 days. Therefore, PLGA is a promising biomaterial to be utilized in sustained-release RNA delivery systems for the treatment of TMJOA. PLGA-based systems in delivering siRNA are usually in conjunction with polyethylenimine (PEI). PEI is a kind of cationic polymeric transfection agents. The incorporation of PEI can protect siRNAs from degradation by extracellular nucleases, and balance the negative charge of siRNAs and thus enhance cellular uptake (Gary et al., 2007). A study evaluated several important parameters for siRNA-loaded PLGA nanoparticles and indicated the necessity of transfection agents in gene silencing (Jensen et al., 2010). PEI can also increase the ratio of encapsulated siRNA, and transfection efficiency of siRNA-PEI-PLGA microparticles was higher than siRNA-PLGA microparticles (Murata et al., 2008; Wang et al., 2017). Some studies develop PLGA-based microparticle formulations to carry anti-inflammatory RNAs to extend their residence time in the TMJ cavity (Mountziaris et al., 2011). Tumor necrosis factor-α (TNF-α) is a kind of pro-inflammatory cytokines, which is expressed highly in the arthritic TMJs (Bi et al., 2022). Mountziaris et al. (2011) developed a TNF-α siRNA-PEI-PLGA microparticles and investigated their release kinetics. This study showed that PEI could decrease burst release of TNF-α siRNA and serve as a porogen to facilitate the process of later release, which provided clues of a sustained release system for the treatment of TMJ degeneration. Fragment crystallizable gamma receptor III (FcγRIII) is a member of the fragment crystallizable (Fc) receptor family, which prefers to bind with immunoglobulin type G (IgG) and then triggers inflammation (Clay et al., 2018). The FcγRIII siRNA-PEI-loaded PLGA microparticles displayed sustained release of FcγRIII siRNA and PEI over 28 days, and these microparticles also decreased the expression levels of inflammatory cytokines in rat TMJs and reduced changes of meal pattern for 9 days (Mountziaris et al., 2012). Another study reported that a single intra-articular injection of PEI- FcγRIII siRNA can downregulate the expression of FcγRIII in the TMJ tissue and decrease the nociceptive response in the TMJOA rats for 2 days (Kramer et al., 2010). However, this study found that the same dosage of naked FcγRIII siRNA and PEI complexed FcγRIII siRNA had the same anti-inflammatory effect on the inflamed TMJs, which suggested PEI might have no improvement on the transfection efficiency of siRNAs. Therefore, future studies are needed to verify the effect of PEI on siRNA delivery PLGA system and the specific transfection protocol for TMJs. Additionally, these studies utilized CFA-induced TMJ inflammation in the rat model, whose duration is relatively short (Feng et al., 2021), and thus the evaluations of controlled release pattern and inflammatory inhibition of siRNA delivery PLGA-based system are limited. Further studied utilizing TMJOA models with long-lasting inflammation are needed to address this limitation.

### 2.3 Chitosan-based system

Chitosan is a linear amino-polysaccharide with good biocompatible and biodegradable properties, and it has a similar chemical structure to glycosaminoglycans which are the main components of cartilage (Kang et al., 2014). Chitosan can be fabricated as fibers, membranes, nanoparticles, and hydrogels. Moreover, chitosan presents many biological properties, such as antibacterial, antifungal, antioxidant, anti-inflammatory, antihyperglycemic, analgesic, and mucoadhesive properties, and thus chitosan-based formulations are widely applied to medicine and dentistry (Derwich et al., 2022). A retrospective cohort clinical study showed three intra-articular injections of chitosan for the patients with TMJOA can increase maximal interincisal opening, reduce pain intensity, and relieve TMJ sounds, which is not better than that of autologous platelet-rich plasma (PRP) (Li et al., 2021). However, complications related to the intra-articular injections, such as pain and swelling in the area of TMJ, are only observed in the patients receiving PRP injection but not in those receiving chitosan injection (Li et al., 2021). These results suggest intra-articular injections of chitosan only is safe but not effective enough for the treatment of TMJOA. Chitosan is usually obtained from chitin in an incomplete deacetylation process, which make chitosan contain acetamide groups and be positively charged in weak acidic solution. Therefore, the cationic chitosan-based system can passively target to cartilage *via* electrostatic attraction, which shows the possibility to develop the drug delivery systems based on chitosan for osteoarthritic joints (Brown et al., 2020).

HA is also a kind of polysaccharides, which is with similar biochemical structures to chitosan. It is known that HA can restore the rheology and visco-elastic properties of the components of joints, present anti-inflammatory and antinociceptive abilities, and protect the cartilage. HA injections, which are called as visco-supplementation treatment, are beneficial to joint stabilization, lubrication, and nutrition, and thus HA injections are proposed as first-line choice for TMJOA (Ferreira et al., 2018). However, HA can be cleared away rapidly from the joint after injection, while frequent injections can result in partial bone necrosis of the articular tubercle and increase the risk of bone degeneration because of repeated microtrauma (Sato and Kawamura, 2006); Talaat et al. (2016) developed a chitosan-based thermosensitive hydrogel for controlled release of HA in the temporomandibular joints. Chitosan aqueous solution is mixed with β-glycerophosphate to prevent immediate gelation and achieve thermosensitive hydrogels, and a Chitosan:β-glycerophosphate ratio of 1:25 is chosen to carry HA in this study. The results show that this chitosan-based thermosensitive hydrogel can retain more than one-third of injected HA in the TMJs after 7 days, which is much more than the controls (injection with HA solution). Therefore, this chitosan-based formulation is a promising controlled release system of HA, and avoid frequent injection of HA for TMJOA therapy. Further studies are required to assess the efficiency of this chitosan-based formulation for longer durations, and optimize release of HA at different time point for better TMJ therapeutics.

### 2.4 Mesoporous silica nanoparticles-based system

Mesoporous silica nanoparticles (MSNs) have gained more and more attention for the promising biomedical applications in the past decade (Tang et al., 2012). Featured with the high surface area and abundant surface functionalization, tailored pore size and volume, and stable framework, MSNs serve as excellent drug drivers with rapid development (Tang et al., 2012; Li et al., 2019). MSNs can be utilized as a local treatment, carrying osteostatin and SOST siRNA to exhibit anti-osteoporotic effects *via* bone marrow injections (Mora-Raimundo et al., 2019). Additionally, MSNs-based system can also be designed and used as a systemic treatment for osteoporosis. For example, MSNs functionalized with alendronate can deliver biomolecules to bone through intravenous administration (Mora-Raimundo et al., 2021).

MSNs with a core-cone structure (MSN-CC) present large pore size and high pore volume, which have demonstrated the high loading capacity of proteins with large molecular weights and showed the ability of cellular delivery of active large proteins (Xu et al., 2015). Li et al. (2019) engineer MSN-CC with PEI modification (MSN-CC-PEI) to load and drive hyaluronan synthase type 2 (HAS2) to synoviocytes in the TMJs. HAS2 is a large-sized protein which produces HA with high molecular weight and maintains the normal functions of synovial fluid (Ishizuka et al., 2019). It is showed that around 82.1% of total MSN-CC-PEI enter and deliver HAS2 into the cells, and HAS2-loaded MSN-CC-PEI can escape from endosome and release HAS2 in cytoplasm of synoviocytes to synthesis HA with high molecular weight (Li et al., 2019). Notably, one-shot administration of HAS2-loaded MSN-CC-PEI maintains the high HAS2 activity and inhibits the synovial inflammation for more than 3 weeks in the rat TMJOA inflammation model, and also shows good ability of TMJ bone defects repairment after 12 weeks in another rat model (Li et al., 2019). This MSN-based nanocarrier offers an efficient and easy-to-use approach for TMJOA management.

### 2.5 Poloxamer micellar system

Poloxamers is a nonionic triblock copolymer consisting of poly (propylene oxide) (PPO) and poly (ethylene oxide) (PEO) arranged in an ABA-like basic structure (i.e., the central hydrophobic units of PPO flanked by two hydrophilic chains of PEO). Physicochemical changes in temperature, pH, or ionic concentration can trigger self-assemble of poloxamers and formation of cross-linked hydrogels by micellar rearrangement. Additionally, different kinds of poloxamers can be associated to form binary systems to develop new carrier systems with different biopharmaceutics properties.

Poloxamer 407, also referred to as Pluronic F-127, has good biocompatibility and is widely used as a drug delivery vehicle (Moura et al., 2020; Liao et al., 2021). It is reported that PEO-PPO-PEO hydrogel Pluronic F127 can load recombinant adeno-associated virus (rAAV) vectors overexpressing the chondrogenic sox9 transcription factor (rAAV-FLAG-hsox9), which form an injectable and thermosensitive therapeutic hydrogel capable of sustained release of rAAV-FLAG-hsox9 (Madry et al., 2020). This rAAV-FLAG-hsox9/PEO-PPO-PEO can repair the cartilage with arranged collagen fibers and protect the subchondral bone plates from bone loss (Madry et al., 2020). Zhang et al. (2021) utilize poloxamer 407 and poloxamer 188 to prepare a glucosamine-encapsulated thermoresponsive hydrogel. The sol–gel transition temperature of this hydrogel is approximately 35°C and this hydrogel also presents a slow release of glucosamine *in vitro*. Intra-articular administration of glucosamine-loaded thermoresponsive hydrogel in rabbits with OA can reduce the degree of swelling, inhibit release of inflammatory factors, and repair the damaged cartilage. In addition, poloxamer 407 is utilized to carry 15d-PGJ_2_ to manage inflammatory pain of TMJOA (Abdalla et al., 2020). It is showed that 15d-PGJ_2_-loaded poloxamer micellar system can relieve TMJ pain at lower concentrations and in a more sustained manner than free 15d-PGJ_2_.

## 3 Transdermal drug delivery systems

**FIGURE 2:**
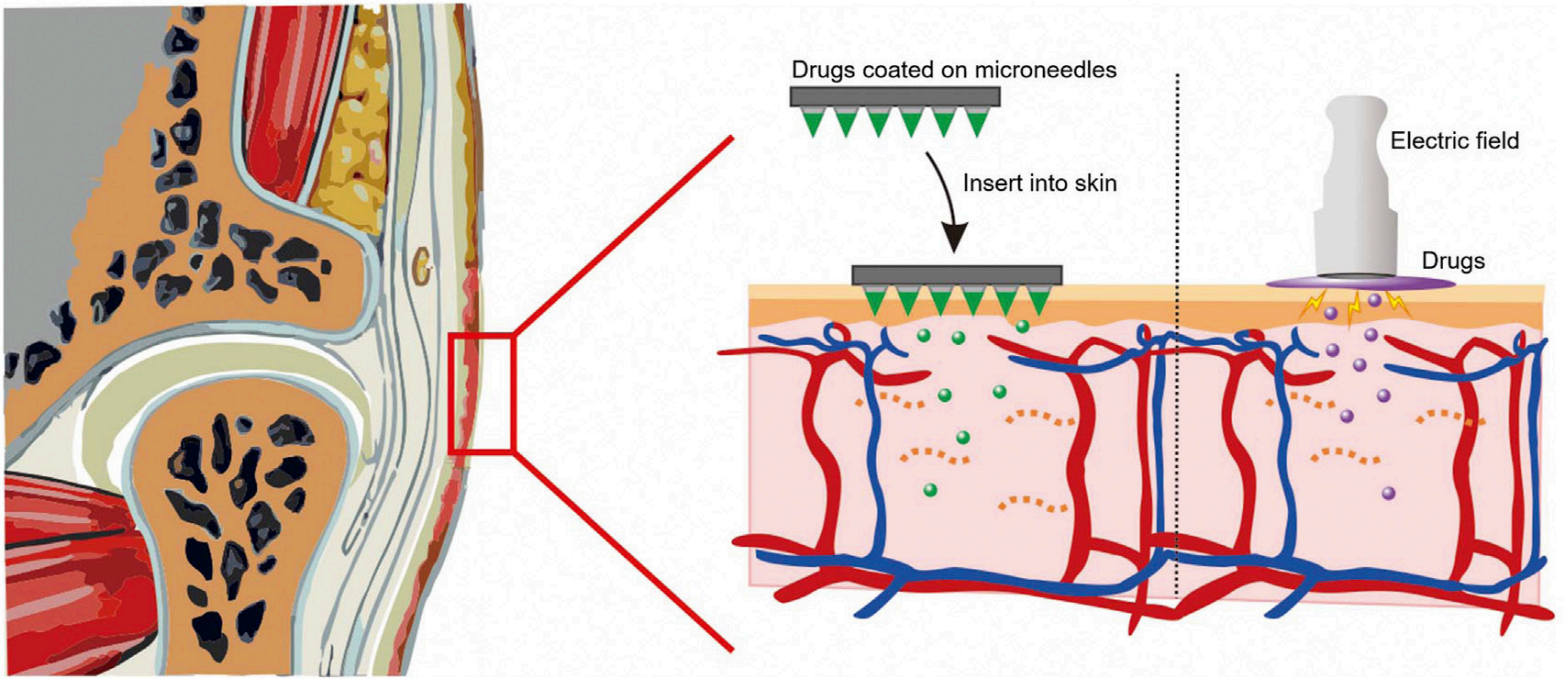
Physical techniques of transdermal drug delivery for TMJOA.

Transdermal drug delivery approaches can avoid first-pass metabolism, control the drug release rates over a prolonged time, and reduce the risk of systemic side effects (Morry et al., 2015). Skin also acts as a reservoir, which enables the diffusion of the penetrated drugs from skin continuously for a longer period and presents an opportunity of lower frequency of administration (Qu et al., 2022). As the stratum corneum (SC) layer of the skin is the capital barrier which limits the penetration of most drugs, many approaches have been adopted to disrupt the SC layer and increase skin permeability, including physical enhancers (iontophoresis, electroporation, sonophoresis, and microneedles), chemical enhancers (alcohol and peptide), and delivery vehicles (spherical nucleic acid and lipoplexes) (Ita 2017; Phatale et al., 2022; Qu et al., 2022). It is reported that transdermal drug delivery approaches enable topical drug delivery to the periarticular tissues of TMJs, which offer a painless and convenient platform to treat TMJOA-associated conditions (Figure 2 and Table 2).

**TABLE 2:** Different transdermal drug delivery systems for TMJOA.

Method	Active molecule	Model	Effects on TMJOA	Duration	Ref
Microneedles	15d-PGJ_2_	Formalin-induced TMJ nociception in rats	Reduce the pro-inflammatory cytokines and nociceptive behavior	Up to 8 h	Macedo et al. (2017)
Microneedles	Tramadol	Formalin-induced TMJ nociception in rats	Reduce the pro-inflammatory cytokines and nociceptive behavior	2 days	Abdalla et al. (2019)
Electroporation	Diclofenac sodium	Patients with arthralgia	Improvement of pain, maximum unassisted opening, and electromyography indices	7 days	Tartaglia et al. (2020)

### 3.1 Microneedles

Microneedles (MNs) patches are comprised of micron-scaled sharp projections, which can overcome SC resistance in a painless manner and transport of drugs into the skin with improved safety, efficacy, and bioavailability (Queiroz et al., 2020; Chen et al., 2022). One study evaluates the influence of MNs on 15d-PGJ_2_ transdermal delivery in a rat model of TMJ pain (Macedo et al., 2017). The results show that 15d-PGJ_2_ cream associated with MNs can hinder the release of pro-inflammatory cytokines and reduce the formalin-induced nociceptive behavior significantly, whose antinociceptive effects are more obvious and last longer than an intra-TMJ injection of 15d-PGJ_2_. Therefore, 15d-PGJ_2_ cream associated with MNs is a promising patient-friendly therapeutic method for pain management related to TMJOA. Similarly, another study coats MNs with tramadol, and finds that tramadol delivery *via* MNs produce more durable antinociceptive effects than intra-TMJ injection of tramadol (Abdalla et al., 2019). However, there is no coating on MN patches for controlling the cargo leakage, which results in burst release of cargoes at the beginning (Wang et al., 2020). This restricts the applications of MN-based drug delivery systems on longitudinal treatment, and thus various controlled delivery nanoparticals are expected to be applied to the MN platform for broadening the biomedical applications.

### 3.2 Electroporation

Electroporation is another physical enhancer used in transdermal drug delivery systems. Electroporation utilizes short (less than 1 s) and high voltage (50–500 V) pulses to cause electropermeabilization and enhance the electrophoretic mobility and molecular diffusivity (Blagus et al., 2013). The temporary aqueous pores in cell membranes alter the electrical conductivity of stratum corneum, and thus the skin barrier is overcome for drug penetration. It is reported that electroporation is utilized to deliver biomolecules of different lipophilicities and molecular weight through enhanced passive diffusion. A clinical study compares the effects of an electroporation-enhanced transdermal application of diclofenac sodium to a conventional intra-articular injection of corticosteroids on TMD associated pain (Tartaglia et al., 2020). The results show that the electroporation in transdermal delivery of diclofenac sodium decreases TMD-related pain levels, improves mouth opening and electromyography to normal values for 1 week after application, while the long-lasting effects are planned to evaluated in future.

## 4 Future outlooks

Oral administration and intra-articular injection are the most widely used routes in the TMJOA treatment. There are some novel drug delivery systems by intra-articular injection which release large or small biomolecules continually and controllable. They can decrease the clearance rate of biomolecules, reduce the inflammatory cytokines release, and promote the repair of cartilage defects effectively (Cao et al., 2021). Although some achievements have been made, there still remains some questions to be answered. For example, 1) can the drug delivery systems by intra-articular injection target more specifically to diseased sites during OA progress; 2) how about the long-term effect and 3) chronic toxicity of these delivery systems, as the studies now usually utilize the OA models with short duration.

By oral administration, the active ingredients reach TMJs *via* the circulatory system, which only allows a small amount of drug to the diseased cartilage and also faces problems including low bioavailability, gastrointestinal injury, and even liver and kidney damage (Raza et al., 2014; Cao et al., 2021). However, few studies have so far focused on the oral administration of drug delivery systems for TMJOA. As oral administration is a convenient and painless route without the need for doctors’ operation, it is significant to design and develop more TMJ-targeted oral drug delivery systems with a sustained release pattern and high bioavailability. Topical drug administration *via* transdermal delivery systems can allow drugs to cross skin barriers and remarkably reduce systemic toxicity and irritation (Li et al., 2021). However, most studies develop transdermal delivery systems for knee OA therapy, while only three studies from two labs utilize MN patches or electroporation to relieve TMJOA-related pain and jaw function restriction. Moreover, various controlled delivery nanoparticals are expected to be applied to the transdermal delivery platforms for broadening the biomedical applications in TMJOA therapy Prausnitz and Langer. (2008).
